# Biogenic Synthesis of Novel Functionalized Selenium Nanoparticles by *Lactobacillus casei* ATCC 393 and Its Protective Effects on Intestinal Barrier Dysfunction Caused by Enterotoxigenic *Escherichia coli* K88

**DOI:** 10.3389/fmicb.2018.01129

**Published:** 2018-06-18

**Authors:** Chunlan Xu, Yu Guo, Lei Qiao, Li Ma, Yiyi Cheng, Alexandra Roman

**Affiliations:** The Key Laboratory for Space Bioscience and Biotechnology, School of Life Sciences, Northwestern Polytechnical University, Xi’an, China

**Keywords:** *Lactobacillus casei*, nanoselenium, mechanism, biosynthesis, probiotic, antioxidant, anticancer, intestinal barrier

## Abstract

Selenium (Se) is an essential element for human and animal health. Biogenic selenium nanoparticles (SeNPs) by microorganism possess unique physical and chemical properties and biological activities compared with inorganic Se and organic Se. The study was conducted to investigate the mainly biological activities of SeNPs by *Lactobacillus casei* ATCC 393 (*L. casei* 393). The results showed that *L. casei* 393 transformed sodium selenite to red SeNPs with the size of 50–80 nm, and accumulated them intracellularly. *L. casei* 393-SeNPs promoted the growth and proliferation of porcine intestinal epithelial cells (IPEC-J2), human colonic epithelial cells (NCM460), and human acute monocytic leukemia cell (THP-1)-derived macrophagocyte. *L. casei* 393-SeNPs significantly inhibited the growth of human liver tumor cell line-HepG2, and alleviated diquat-induced IPEC-J2 oxidative damage. Moreover, *in vivo* and *in vitro* experimental results showed that administration with *L. casei* 393-SeNPs protected against Enterotoxigenic *Escherichia coli* K88 (ETEC K88)-caused intestinal barrier dysfunction. ETEC K88 infection-associated oxidative stress (glutathione peroxidase activity, total superoxide dismutase activity, total antioxidant capacity, and malondialdehyde) was ameliorated in *L. casei* 393-SeNPs-treated mice. These findings suggest that *L. casei* 393-SeNPs with no cytotoxicity play a key role in maintaining intestinal epithelial integrity and intestinal microflora balance in response to oxidative stress and infection.

## Introduction

Selenium (Se) as an essential trace element plays a fundamental role in human and animal body. Se is involved in the body’s metabolism and exerts antioxidative, antiaging, antitumor, and immune regulatory functions (Rayman, 2006; Schomburg, 2017). Selenoproteins and enzymes containing selenocysteine are the main forms of Se which exerts antioxidant activity. Se is the key cofactor of glutathione peroxidases (GPx) and thioredoxin reductases. The chemical forms of Se in nature mainly include inorganic Se such as selenite (Na_2_SeO_3_) and selenate (Na_2_SeO_4_), elemental (0), and organic Se such as selenomethionine/selenocysteine, etc. (Biswas et al., 2011; Li et al., 2014). Sodium selenite is the toxic form of the trace element Se. However, selenium nanoparticles (SeNPs) transformed from sodium selenite exhibit low or no cytotoxicity. Up to now, SeNPs with unique properties and diverse functions have wide applications in medicine, therapeutic, biosensors, and environmental remediation (Wadhwani et al., 2016). Therefore, it is meaningful to establish green, efficient, and low-cost approaches for transforming selenite to SeNPs with various activities.

Selenium nanoparticles are usually synthesized by physical, chemical, and biological methods. Physical methods mainly include UV radiation, laser ablation, and hydrothermal techniques (Quintana et al., 2002; Iranifam et al., 2013; Van Overschelde et al., 2013). Ascorbic acid, glucose, sulfur dioxide, and sodium dodecyl sulfate, etc. are usually used for the chemical synthesis of SeNPs, which are involved in lines of catalytic reduction (Hong Lin et al., 2004; Zhang et al., 2010; Dwivedi et al., 2011). Previous researches indicate that certain bacteria (Cui et al., 2016), fungi (Vetchinkina et al., 2013), and plants (Salunke et al., 2014) can mediate the biological synthesis of SeNPs. However, in the process of preparation of SeNPs, unsafe factors brought by physical and chemical methods limit the development and wide application of SeNPs in food, biomedicine, and feed additives (Iranifam et al., 2013). However, biogenic synthesis of SeNPs usually applies some safe, low-cost, eco-friendly, and non-toxic materials (Salunke et al., 2014; Singh et al., 2015; Cui et al., 2016). Furthermore, biogenic synthesis of SeNPs by probiotics is large-scale, green and safe, low cost, high efficiency, and without seasonal restrictions. Therefore, this method attracted wide interests and attention due to above advantages. Se-enriched probiotic bacteria could provide a better alternative as a dietary supplement due to the double efficacy of Se and probiotics (Pieniz et al., 2011; Saini et al., 2014). *Lactobacillus casei* (*L. casei*) can synthesize lactomicroselenium particles with a size of 85–200 nm (Nagy et al., 2016). *L. casei* ATCC 393 (*L. casei* 393) is an important probiotic bacteria and usually applied in fermented dairy products and functional foods (Kourkoutas et al., 2006; Sidira et al., 2010, 2013, 2014). Recent researches indicated that *L. casei* 393 possesses immunomodulatory, anti-inflammatory, and anti-tumor activities (Sidira et al., 2010; Tiptiri-Kourpeti et al., 2016). However, up to now, the mechanism of probiotics-mediated synthesis of SeNPs is still unclear. Moreover, the potential biological activity and application effect of SeNPs-enriched *L. casei* 393 (*L. casei* 393-SeNPs) need further investigation.

In the present study, SeNPs were synthesized using fermentation technology based on probiotic bacteria strain *L. casei* 393 in a simple, low-cost green way. The generated SeNPs inside bacteria were characterized by Flame Emission Atomic Absorption Spectrometer, Transmission Electron Microscopy (TEM), and Energy Dispersive X-ray (EDX). Furthermore, the antioxidant and anti-hepatocarcinoma activity, and protective effect of *L. casei* 393-SeNPs on the Enterotoxigenic *Escherichia coli* K88 (ETEC K88)-induced intestinal epithelial barrier dysfunction were evaluated by *in vitro* and *in vivo* experiments.

## Materials and Methods

### Bacterial Strains, Cell Line, and Reagents

*Lactobacillus casei* ATCC 393 strain was purchased from the National Collection of Agricultural and Industrial Microorganisms (Beijing, China). ETEC K88 and porcine jejunal cell line IPEC-J2 were kindly donated by Prof. Yizhen Wang (Zhejiang University, China). Human acute monocytic leukemia cell line (THP-1) was kindly donated by associate Prof. Dongyan Shao. Human normal epithelial cell NCM460 cell line was purchased from Cell Resource Center, Shanghai Institute, Chinese Academy of Sciences. deMan, Rogosa, and Sharpe (MRS) broth, sodium selenite and fluorescein isothiocyanate (FITC)-Dextran (FITC-Dextran), and phorbol-12-myristate-13 acetate (PMA) were purchased from Sigma (St. Louis, MO, United States). Luria-Bertani (LB) broth was purchased from Oxoid (Basingstoke, United Kingdom). Dulbecco’s Modified Eagle’s medium/Ham’s F-12 (DMEM/HF12) medium, penicillin/streptomycin, 0.25% trypsin-EDTA, and fetal bovine serum (FBS) were purchased from Life Technologies (Grand Island, NY, United States). Enzyme-linked immunosorbent assay (ELISA) kits for tumor necrosis factor-α (TNF-α), interferon-γ (IFN-γ), interleukin-1β (IL-1β), and vasoactive intestinal peptide (VIP) were purchased from R&D Systems (Minneapolis, MN, United States). Primary antibodies against zonula occludens 1 (ZO-1), occludin, claudin-1, brain-derived neurotrophic factor (BDNF), tropomyosin receptor kinase B (TrkB) and glyceric acid phosphate dehydrogenase (GADPH), and peroxidase-conjugated secondary antibodies were obtained from Santa Cruz Biotechnology, Inc. (Santa Cruz, CA, United States). Alkaline phosphatase (ALP), GPx, total superoxide dismutase (T-SOD), total antioxidant capacity (T-AOC), and malondialdehyde (MDA) assay kits were purchased from Nanjing Jiancheng Bioengineering Institute (Jiangsu, China). Bicinchoninic acid (BCA) protein assay kit was purchased from Solarbio Life Sciences Co. (Beijing, China). Hoechst 33342 staining kit and Cell Counting kit-8 (CCK-8) were purchased from Beyotime Biotechnology (Shanghai, China).

### Preparation of *L. casei* 393-SeNPs

*Lactobacillus casei* ATCC 393-SeNPs was prepared according to the following steps. Briefly, *L. casei* 393 was grown in MRS broth at 37°C for 24 h under anaerobic conditions without shaking. Then the medium was cultivated with 200 μg/ml of sodium selenite at 37°C for continuous 24 h. During the fermentation process, color of the broth was observed. At the end of fermentation, culture medium was centrifuged at 12,500 × *g* at 4°C for 10 min. Then pellets were washed twice with phosphate buffer solution (PBS, pH 7.4) and resuspended in 1 ml PBS. Parts of samples were filtered through a 0.22-μm millipore filter. The color of supernatant and precipitate was also observed.

### Detecting the Concentration and Size of Elemental Nanoselenium in *L. casei* 393

The final Se concentration of the enriched-Lactonanoselenium *L. casei* 393 samples was determined by Flame Emission Atomic Absorption Spectrometer (Therm ICE 3000) and Atomic Fluorescence Spectrometer (PSA Thermo-Fisher Excalibur, Waltham, MA, United States). Firstly, 2 ml fermentation broth was centrifuged at 12,500 × *g* at 4°C for 10 min. The pellet was digested in the mix of nitric acid and hydrogen peroxide (v/v, 9:1), and heated at 120°C for 60 min. After that the digested samples were filtered and adjusted to 10 ml with distilled water. Meanwhile, standard solution of Se was prepared. The concentration of Se in samples was calculated according to the standard curve method. TEM of Model JEM-1230 (JEOL, Tokyo, Japan) has been used to visualize and measure the size of Se particles in *L. casei* 393 under standard operating conditions. Ultraviolet–Visible (UV–Vis) Absorption Spectrophotometer (U-3310, HITACHI, United States) was used to observe the absorption spectrum of *L. casei* 393-SeNPs. *L. casei* 393 was used as control.

### Cytotoxicity Analysis of *L. casei* 393-SeNPs *in Vitro*

NCM460, IPEC-J2, and THP-1 cells were used to evaluate the cytotoxicity of *L. casei* 393-SeNPs. HepG2 and THP-1 cells were grown at 37°C in DMEM containing high glucose (Gibco, Carlsbad, NM, United States) supplemented with 10% FBS (Gibco, Carlsbad, NM, United States), 1% penicillin–streptomycin (Sigma, St. Louis, MO, United States). IPEC-J2 cells were cultured in DMEM/Ham’s F-12 (1:1) medium supplemented with 10% FBS and 1% antibiotic mixture (100 U/ml penicillin and 100 U/ml streptomycin). Living cell viability and the cytotoxicity of *L. casei* 393-SeNPs were evaluated by CCK-8 kit. Briefly, NCM460 or IPEC-J2 cells (1 × 10^5^) were seeded in 96-well plates for 12 h. For induction of THP-1 differentiation, 2 × 10^6^ CFU/ml cells were seeded in the presence of 60 nM PMA and incubated for 48 h. Then the cells were treated with *L. casei* 393-SeNPs containing different concentration of Se for another 12 h. Finally, 10 μl CCK-8 was added to each well and incubated with cells for 2 h. Absorbance was detected at 450 nm using a microplate reader (Bio-Rad Laboratories, Hercules, CA, United States).

### Anticancer and Antioxidative Activity Analysis of *L. casei* 393-SeNPs

Moreover, we further investigated the anticancer and antioxidant activity of *L. casei* 393-SeNPs. Briefly, IPEC-J2 cells (1 × 10^5^) were seeded in 6-well plates for 12 h. Then IPEC-J2 cells were treated with 100 μM diquat, *L. casei* 393-SeNPs containing 4 μg/ml Se or DMEM/Ham’s F-12 (1:1) medium without FBS and antibiotics for another 12 h. Cell morphology was observed by optical microscope. Moreover, the expression levels of tight junction proteins (ZO-1, occludin, and claudin-1) were measured by western blot. The level of MDA was determined by MDA kit. HepG2 cells (1 × 10^5^) were incubated in six-well plates for 24 h. The treatment group was cultivated with *L. casei* 393-SeNPs containing 8 μg/ml Se or high glucose-DMEM medium without FBS and antibiotics for another 12 h. Then cells were stained with 6 μg/ml Hoechst 33342 at 37°C for 10 min. For each group, cell morphology and count were observed under an inverted fluorescence microscope (Leica DMIL, Germany).

### Protection of IPEC-J2 by *L. casei* 393-SeNPs Against ETEC K88 Challenge

*Lactobacillus casei* ATCC 393 was grown in MRS at 37°C overnight under 1.2 mM sodium selenite stress anaerobic conditions and without shaking. ETEC K88 was grown in LB broth at 37°C overnight with vigorous shaking at 120 rpm. The optical density (OD) of experimental strain was measured by Spectrophotometer. The bacteria were harvested by centrifugation at 5000 × *g* at 4°C for 10 min and washed with PBS (pH 7.4). The bacterial pellet was responded in antibiotic-free cell culture medium and diluted to the desired concentration using hemocytometer under light microscope to count the bacterial numbers. At the same time, the supernatant of *L. casei* 393-SeNPs and ETEC K88 was filtered through a 0.22-μm filter and diluted (1:10) with antibiotic-free cell culture medium. IPEC-J2 cells (1.0 × 10^6^ cells per filter) were seeded into 6-well Transwell collagen-coated PTFE filter. After the cells were completely differentiated, cells were treated under the following conditions: (1) medium (control); (2) ETEC K88 (1 × 10^7^ CFU/ml) infection alone (ETEC K88); (3) pre-incubation simultaneous incubation with 2 ml of medium containing *L. casei* 393-SeNPs for 2 h prior to addition of ETEC K88 (1 × 10^7^ CFU/ml) (*L. casei* 393-SeNPs + ETEC K88). IPEC-J2 cells and medium were harvested at 3 h after ETEC K88 challenge and stored at -80°C until assayed. The concentration of ALP in medium was detected by ALP ELISA kit according to the manufacturer’s instruction. Total RNA was extracted from IPEC-J2 cells using Trizol reagent (Invitrogen, Carlsbad, CA, United States). Real-time PCR was performed in LightCycler480II system (Roche, Mannheim, Germany) using a SYBR Premix ExTaq^TM^II qPCR Kit (TaKaRa). mRNA levels of Toll-like receptor 2 (TLR2), TLR4, TLR9, nucleotide-binding oligomerization domain 1 (NOD1), and NOD2 were quantified. β-Actin was used as a reference transcript. Primers sequences were shown in **Table 1**. Primers were designed using Primer premier 5.0 software and were synthesized by Sangon Biotech Co. (Shanghai, China). All samples were tested in duplicate and relative mRNA abundances of the target genes were determined using the 2^-ΔΔCt^ method as ΔCT = CT (target gene)-CT (β-actin), ΔΔCt = ΔCt (targeted group) ΔCt (control group). IPEC-J2 cells were homogenized with 200 μl RIPA lysis buffer (Solarbio Life Sciences Biotech Co., Beijing, China) and centrifuged at 12,000 × *g* for 15 min at 4°C to collect the supernatants. The expression levels of occludin and ZO-1 were detected by western blot. GADPH was used as a reference control. The OD of each band was quantified by densitometric analysis using Quantity One software (Bio-Rad Laboratories, Hercules, CA, United States). Results are presented as the abundance of each target protein relative to GADPH in the same samples.

**Table 1:** Sequences of oligonucleotide primers used for PRRs signaling pathway genes mRNA levels analysis.

Gene product^a^		Primer	Product size (bp)
		
	Direction^b^	Sequence (5′–3′)	
GADPH	F	CCAGAACATCATCCC TGCTT	229
	R	GTCCTCAGTGTAGCCCAGGA	
TLR2	F	TCACTTGTCTAACTTATCATC CTCTTG	162
	R	TCAGCGAAGGTGTCATTATTGC	
TLR4	F	GCCATCGCTGCTAACA TCATC	108
	R	CTCATACTCAAAGATACAC CATCGG	
TLR9	F	GTGGAACTGTTTTGGCATC	199
	R	CACAGCACTCTGAGCTTTGT	
NOD1	F	ACCGATCCAGTGAGC AGATA	140
	R	AAGTCCACCAGCTCCATGAT	
NOD2	F	GAGCGCATCCTCTTAACTTTCG	66
	R	ACGCTCGTGATCCGTGAAC	


*^*a*^GADPH, glyceraldehyde-3-phosphate dehydrogenase; TLR, toll-like receptor; NOD, nucleotide-binding oligomerization domain. ^*b*^F, forward; R, reverse.*

### Protective Effect of *L. casei* 393-SeNPs on C57BL/6 Mice Challenged by ETEC K88 Challenge

This animal experiment was approved by the Institutional Animal Care and Use Committee of the Northwestern Polytechnical University (Permit Number: 20161005) and conducted in accordance with the National Institutes of Health guidelines for the care and use of experimental animals. Forty adult male C57BL/6 mice (19 ± 2 g) were purchased from the Experimental Animal Center of Xi’an Jiaotong University. During the whole experimental period, mice were maintained at the Animal Experimental Center of Northwestern Polytechnical University at room temperature of 25°C, relative humidity of 50%, and a 12 h light and dark cycle. In the experiment, healthy male C57BL/6 mice were assigned randomly to four groups: normal control group (MRS medium and LB medium), model group (MRS medium and ETEC K88 culture medium), *L. casei* 393 protective group (*L. casei* 393 culture medium and ETEC K88 culture medium), and *L. casei* 393-SeNPs protective group (*L. casei* 393-SeNPs culture medium and ETEC K88 culture medium), with 10 animals in each group. The experimental duration lasts for 14 days. The mice in *L. casei* 393 protective group were orally dosed with 500 μl (10^9^ CFU/ml) of inoculums of *L. casei* 393. The mice in *L. casei* 393-SeNPs protective group were orally dosed with 500 μl (10^9^ CFU/ml) of inoculums of enriched SeNPs-*L. casei* 393 (10^9^ CFU/ml) with 22.76 μg/ml Se. The mice in the other groups were orally given the same volume of MRS broth per day. On days 8, 10, and 12, other groups were given 500 μl (10^9^ CFU/ml) of inoculums of ETEC K88 beside control group administrated with LB broth. Diarrhea and bloody stool were observed. Moreover, before sampling, there are no food and water on day 15 for 7 h, and three mice were orally given 40 mg/ml FITC-Dextran for 4 h. Intestinal morphology was evaluated by hematoxylin–eosin (HE) staining. Serum MDA levels, T-AOC, T-SOD, and GPx activities were analyzed using the thiobarbituric acid method according to the kit protocols. The levels of TNF-α, IFN-γ, IL-1β, and VIP in serum were determined by ELISA kits. The expression of occludin, ZO-1, and claudin in ileum tissues, and BNDF and TrKB expression levels in brain were detected by western blot. Microbiome of ileum and cecum content was analyzed by high-throughput sequencing techniques.

### Statistical Analysis

Data were analyzed by one-way analysis of variance (ANOVA) or Student’s *t*-test (SPSS19.0, Chicago, IL, United States). All data were shown as mean ± standard error of mean (SEM), and *P* < 0.05 indicated significant difference in statistics.

## Results

### Characterization and Localization of SeNPs in *L. casei* 393

**FIGURE 1:**
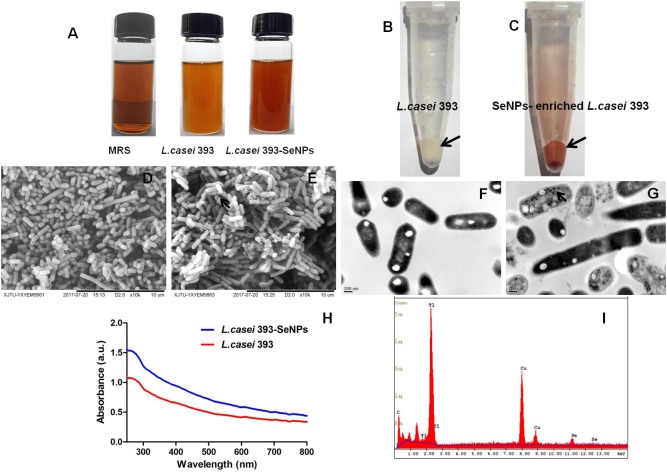
Preparation and characterization of selenium nanoparticles (SeNPs)-enriched *L. casei* 393. **(A)** Appearance of MRS or *L. casei* 393 cultivated with or without Na_2_SeO_3_ at 30°C for 24 h. **(B)** Visible white color of *L. casei* 393. **(C)** *L. casei* 393-SeNPs appear distinct bright red color. **(D)** SEM images of *L. casei* 393. **(E)** SEM images of *L. casei* 393-SeNPs. **(F)** TEM image of *L. casei* 393. **(G)** TEM image of *L. casei* 393-SeNPs. **(H)** Ultraviolet–visible (UV–Vis) absorption spectrum of *L. casei* 393-SeNPs and *L. casei* 393. **(I)** EDX image of *L. casei* 393-SeNPs.

According to the apparent color changes in the culture medium with or without sodium selenite shown in **Figure 1A**, we found that *L. casei* 393 culture medium presented creamy yellow. However, *L. casei* 393 culture medium with sodium selenite exhibited distinct bright red color. As shown in **Figures 1B,C**, *L. casei* 393 and *L. casei* 393-SeNPs present white and red color, respectively, which indicates that the probiotic *L. casei* 393 possessed the ability to transform the toxic, colorless selenite to the non-toxic, red, insoluble elemental form of Se (Se0). SEM images showed that SeNPs-enriched *L. casei* 393 tended to be aggregated compared with the control group (**Figures 1D,E**). TEM images showed that SeNPs with size of 50–80 nm mainly distributed within the *L. casei* 393 (**Figure 1G**). The cytoplasm of *L. casei* 393 was homogeneous and without black particles inside bacteria (**Figure 1F**). As shown in **Figure 1H**, the protein absorption peak appeared at 280 nm concomitantly. EDX Spectrum was usually used to measure the Se distribution. The result showed that Se atom signal was appeared in the EDX spectrum, accounting for about 0.48% of the total component elements (**Figure 1I**).

### Cytotoxicity of *L. casei* 393-SeNPs

**FIGURE 2:**
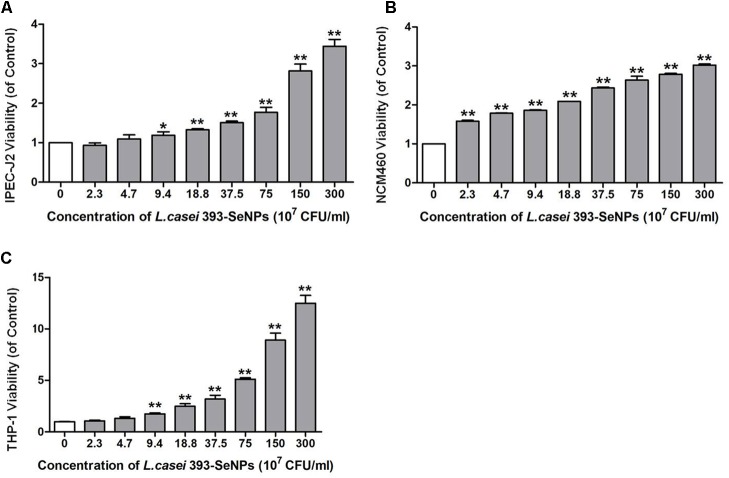
Cytotoxicity of *L. casei* 393 and *L. casei*-SeNPs on **(A)** IPEC-J2, **(B)** NCM460, and **(C)** THP-1. NCM460 and IPEC-J2 cells (1 × 10^5^) were seeded in 96-well plates for 12 h. For induction of THP-1 differentiation, 2 × 10^6^ CFU/ml cells were seeded in the presence of 60 nM phorbol-12-myristate-13 acetate (PMA) and incubated for 48 h. Then cells were treated with *L. casei* 393-SeNPs containing different concentration of Se for another 12 h. Finally, 10 μl CCK-8 was added to each well and incubated with cells for 2 h. Absorbance was detected at 450 nm using a microplate reader. All data were presented as mean ± SEM (*n* = 8). *L. casei* 393-SeNPs vs. the control, ^∗^*P* < 0.05, ^∗∗^*P* < 0.01.

Effect of *L. casei* 393-SeNPs on the IPEC-J2, NCM460, and THP-1 viability was shown in **Figure 2**. *L. casei* 393-SeNPs in the test concentration range did not exert inhibitory effect on the tested cells. Moreover, *L. casei* 393-SeNPs promoted the growth and proliferation of IPEC-J2, NCM460, and THP-1 cells.

### Protective Effect of *L. casei* 393-SeNPs on ETEC K88 Induced Intestinal Barrier Function Damage

**FIGURE 3:**
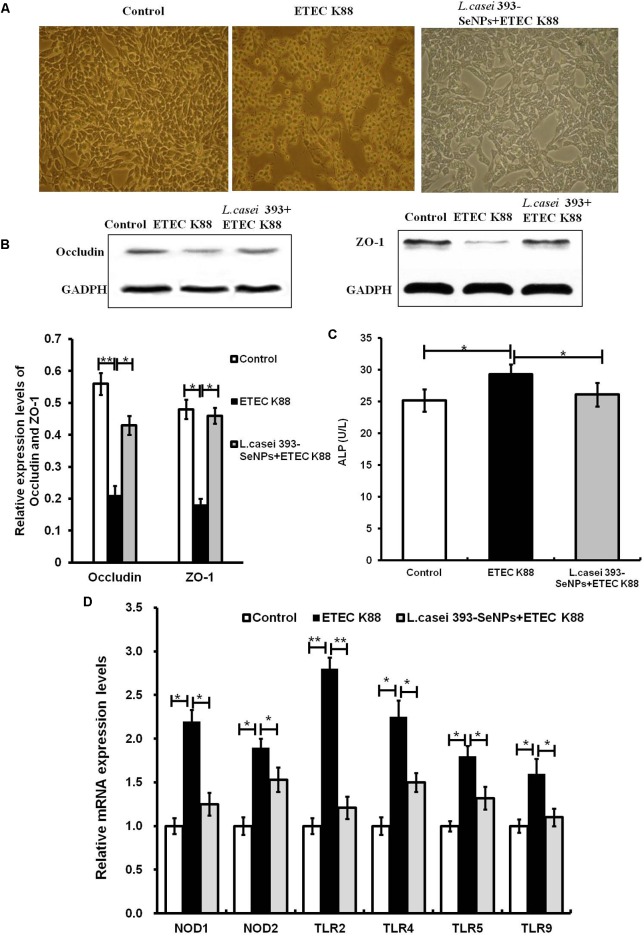
Protective effect of *L. casei*-SeNPs on the IPEC-J2 challenged by ETEC K88. **(A)** Impact of ETEC K88 with or without *L. casei* 393-SeNPs pretreatment on the morphology of IPEC-J2. **(B)** Effect of ETEC K88 with or without *L. casei* 393-SeNPs pretreatment on the occludin and ZO-1 expression. **(C)** Effect of ETEC K88 with or without *L. casei* 393-SeNPs pretreatment on the content of ALP in the cell medium. **(D)** Effect of ETEC K88 with or without *L. casei* 393-SeNPs pretreatment on the PRRs signaling pathway analyzed by western blotting. All data were presented as mean ± SEM (*n* = 8). ^∗^*P* < 0.05; ^∗∗^*P* < 0.01.

As shown in **Figure 3**, it is observed from microscopic morphology that ETEC K88 exerted dramatically toxic effect on IPEC-J2 cells, which appear obvious deformation, shrinkage, and a large number of cell apoptosis. However, pretreatment of *L. casei* 393-SeNPs on IPEC-J2 cells for 2 h significantly antagonized toxic effect of ETEC K88 on IPEC-J2 cells. In addition, pretreatment of *L. casei* 393-SeNPs significantly down-regulated the mRNA levels of TLR2, TLR4, TLR9, NOD1, and NOD2, and reduced the activity of ALP in the culture medium when compared with the ETEC K88 treatment alone. Moreover, compared with treatment of ETEC K88 alone, pretreatment of *L. casei* 393-SeNPs up-regulated the protein expression levels of occludin and ZO-1.

### Antitumor and Antioxidant Properties of *L. casei* 393-SeNPs *in Vitro*

**FIGURE 4:**
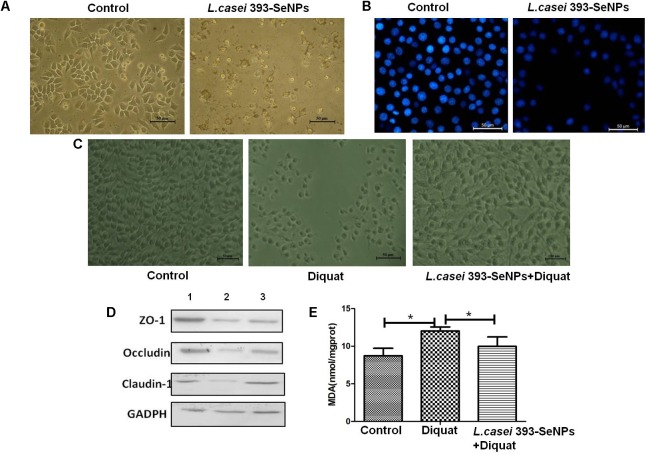
The antitumor and antioxidant activities of *L. casei* 393-SeNPs. **(A)** Suppressive effect of *L. casei* 393-SeNPs on HepG2 cells growth. **(B)** Viability of HepG2 cells cultured with *L. casei* 393-SeNPs containing 8 μg/ml Se. **(C)** Protective effect of *L. casei* 393-SeNPs containing 4 μg/ml Se pretreatment on the IPEC-J2 challenged by diquat. **(D)** The expression levels of different tight junction proteins in IPEC-J2 from each experimental groups analyzed by western blotting. 1, Control; 2, diquat; 3, *L. casei* 393-SeNPs + diquat. **(E)** MAD levels in cultural medium. All data were presented as mean ± SEM (*n* = 8). ^∗^*P* < 0.05. MDA, malondialdehyde; ZO-1, zonula occludens 1.

Compared with the control group, administration with *L. casei* 393-SeNPs significantly inhibited the growth of HepG2 with a large number of cells apoptosis (as shown in **Figures 4A,B**). The antioxidant activity of *L. casei* 393-SeNPs was measured via establishment of diquat-induced oxidative damage on IPEC-J2 cells. As shown in **Figures 4C,E**, administration with 1 mM diquat resulted in the apoptosis of IPEC-J2 and lots of cellular debris were observed. However, treatment with *L. casei* 393-SeNPs along with diquat significantly alleviated cytotoxicity of diquat.

Moreover, as a shown in **Figure 4D** when compared with the control group, 1 mM diquat significantly down-regulated the expression levels of tight junction proteins (ZO-1, occluding, and claudin-1). However, administration with *L. casei* 393-SeNPs significantly relieved down-regulation of ZO-1, occluding, and claudin-1 protein expression levels caused by diquat.

### Anti-infective Activities of *L. casei* 393-SeNPs *in Vivo*

**FIGURE 5:**
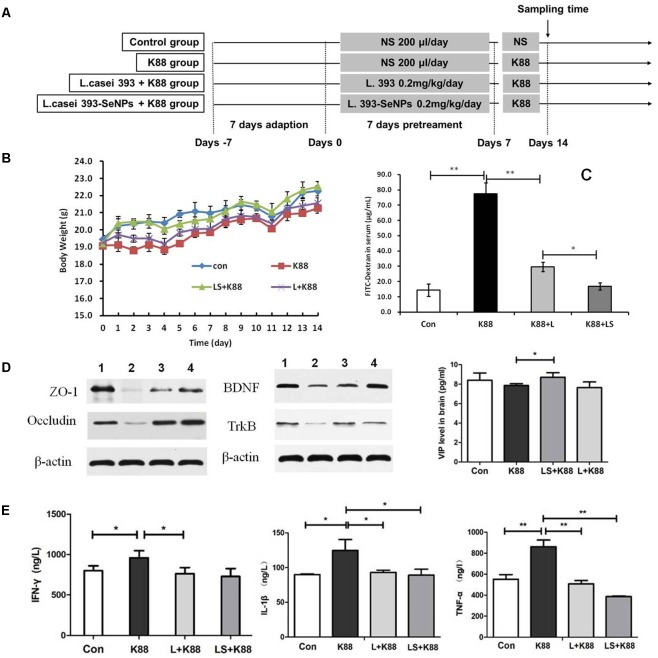
Protective effect of *L. casei* 393-SeNPs on the C57BL/6 mice challenged by ETEC K88. **(A)** The scheme of experiment *in vivo*. **(B)** The change of weight during the whole experiment. **(C)** Modulatory effect of *L. casei* 393-SeNPs on the C57BL/6 mice challenged by ETEC K88. FITC-dextran was used as the indicative material. **(D)** The expression levels of tight junction proteins (ZO-1 and occludin) in ileum, and BDNF and TrkB in brain from the different experiment rats. 1, Control; 2, K88; 3, L+K88; and 4, LS+K88. **(E)** The serum levels of IFN-γ, IL-1β, and TNF-α, and the level of VIP in brain determined by ELISA. All data were presented as mean ± SEM (*n* = 8), ^∗^*P* < 0.05, ^∗∗^*P* < 0.01. K88 means the rats orally administrated with ETEC K88 only. LS + K88 means the rats pretreated with *L. casei* 393-SeNPs before administration with ETEC K88. L + K88 means the rats pretreated with *L. casei* 393 before administration with ETEC K88. ZO-1, zonula occludens 1; BDNF, brain-derived neuotrophic factor; TrkB, tropomyosin receptor kinase B.

**FIGURE 6:**
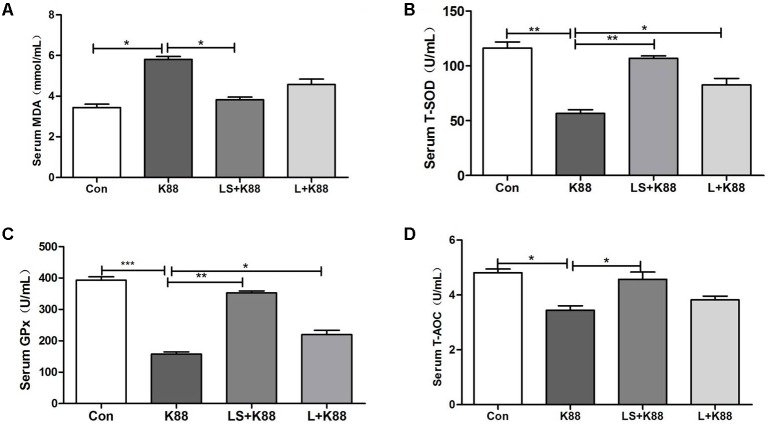
Effects of *L. casei* 393 and *L. casei 393*-SeNPs on oxidative stress in mice challenged by ETEC K88. Serum MDA **(A)**, T-SOD **(B)**, GPx **(C)**, and T-AOC **(D)** levels in male C57BL/6 mice were measured by corresponding kits. Data represent mean ± SEM (*n* = 8), ^∗^*P* < 0.05, ^∗∗^*P* < 0.01, ^∗∗∗^*P* < 0.001. K88 means the rats orally administrated with ETEC K88 only. LS + K88 means the rats pretreated with *L. casei* 393 before administration with ETEC K88. LS means the rats treated with *L. casei* 393 only. MDA, malondialdehyde; GPx, glutathione peroxidase; T-SOD, total superoxide dismutase; T-AOC, total antioxidant capacity.

**FIGURE 7:**
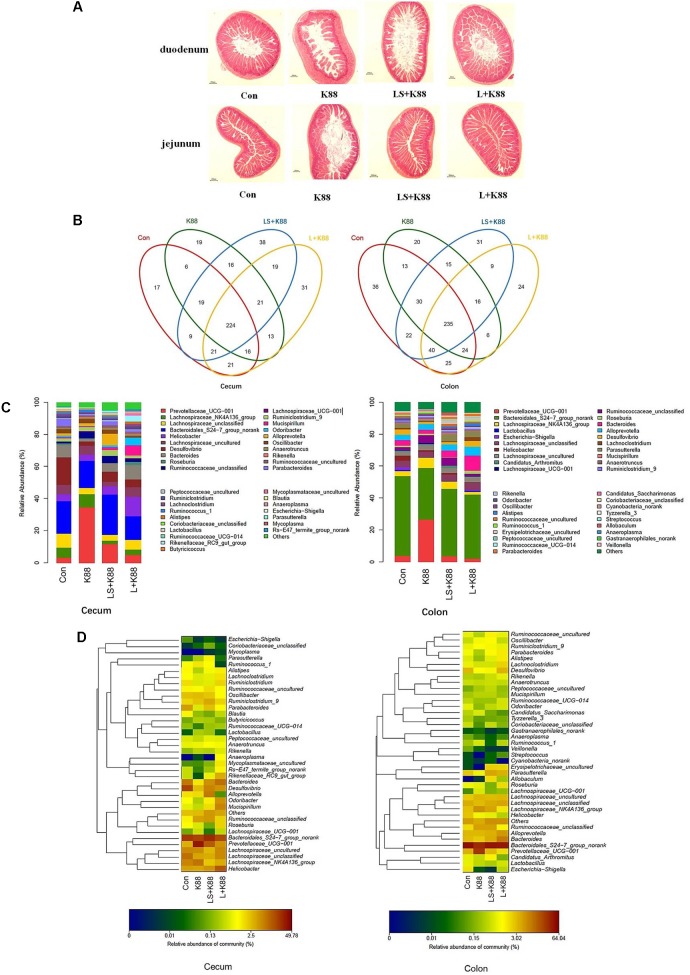
Modulatory effects of *L. casei* 393 and *L. casei 393*-SeNPs on the intestinal morphology and microbiome in mice challenged by ETEC K88. **(A)** The histology of duodenum and jejunum. **(B)** Shared OUT analysis of the different libraries. Venn diagram showing the unique and shared OTUs (3% distance level) in the different libraries. **(C)** Bacterial composition of the different communities. Relative read abundance of different bacterial genus within the different communities. Sequences that could not be classified into any known group were assigned as “unclassified bacteria.” **(D)** Bacterial distribution for the cecum and colon libraries from different treatments. Double hierarchical dendrogram showing the bacterial distribution among the samples. The bacterial phylogenetic tree was calculated using the neighbor-joining method and the relationship among samples was determined by Bray distance and the complete clustering method. The heatmap plot depicts that the relative percentage of each bacterial family are depicted by color intensity with the legend indicated at the bottom of the figure. Clusters based on the distance of the four experimental treatments along the *X*-axis and the bacterial families along the *Y*-axis are indicated in the upper and left of the figure, respectively. K88 means the rats orally administrated with ETEC K88 only. LS + K88 means the rats pretreated with *L. casei* 393-SeNPs before administration with ETEC K88. L + K88 means the rats pretreated with *L. casei* 393 before administration with ETEC K88.

The experimental scheme was shown in **Figure 5A**. As shown in **Figure 5B**, the body weight gradually increased during the whole experimental period. The body weight of mice from the *L. casei* 393-SeNPs or *L. casei* 393 group was higher than that of ETEC K88 group. FITC-Dextran was usually used to investigate the intestinal barrier permeability. The result showed that serum FITC-Dextran level of the ETEC K88 group was higher than that of other groups (**Figure 5C**). When compared with the ETEC K88 group, previously oral administration with *L. casei* 393-SeNPs or *L. casei* 393 obviously relieved the decrease of protein expression levels of ZO-1, occludin, BDNF, and NTRK2 caused by ETEC K88 (**Figure 5D**). Moreover, administration with ETEC K88 significantly increased the serum levels of IFN-γ, IL-1β, and TNF-α compared to the control group (**Figure 5E**). However, pretreatment with *L. casei* 393-SeNPs or *L. casei* 393 significantly inhibited the increase of IFN-γ, IL-1β, and TNF-α in serum. Pretreatment with *L. casei* 393-SeNPs remitted the increase of brain VIP level caused by ETEC K88 (**Figure 5F**). Moreover, ETEC K88 challenge caused the oxidative stress of experimental mice. Administration of *L. casei* 393-SeNPs significantly elevated serum GPx, T-SOD, and T-AOC activity, and reduced serum MDA levels compared with the ETEC K88 group (**Figures 6A–D**). In addition, compared with the control group, ETEC K88 treatment resulted in the morphology change, and shorter intestinal villus with disordered arrangement were observed via HE staining. However, the intestinal villus arranged neatly in the previously administration with *L. casei* 393-SeNPs group compared with those in the ETEC K88-treated alone group (**Figure 7A**). Moreover, administration with ETEC K88, *L. casei* 393-SeNPs, or *L. casei* 393 significantly affected the bacterial composition and diversity in cecum and colon content. The OTU composition and abundance were relatively similar between the colon and cecum content in the same group. Each experimental group in cecum and colon shared 224 and 235 OTUs (**Figure 7B**). As shown in **Figures 7C,D**, compared with the normal control, ETEC K88 treatment significantly increased the abundance of *Prevotellaceae*_UCG-001 and *Ruminococcaceae*_unclassified in cecum and colon content, and decreased *Lachnospiraceae*_unclassified and *Lachnospiraceae*_uncultured abundance in cecum content. Moreover, compared to the other experimental groups, ETEC K88 treatment significantly decreased the abundance of *Lactobacillus* in colon. On the contrary, *L. casei* 393-SeNPs treatment significantly alleviated the change of the above bacteria.

## Discussion

Recently, probiotics have acquired considerable significance due to their health beneficial properties. Probiotics can provide benefits to the host gut through a diverse set of mechanism that includes competitive exclusion of pathogens, production of antimicrobial compounds, enterotoxin inactivation, modulation of host immune responses, and maintenance of intestinal barrier integrity (Dubreuil, 2017). Its beneficial properties make it a commendable carrier. Therefore, current study was aimed to investigate the microbial transformation ability of sodium selenite to SeNPs by *L. casei* 393 and the main biological activities of *L. casei* 393-SeNPs.

Selenium as an essential element is closely related to human and animal health. In general, Se must be exogenously supplied in order to meet requirement of human and animal health. Sodium selenite is a common form of supplementation. The toxicity order of different Se species was: selenate > selenite > nanoSe > lactomicroSe (Nagy et al., 2016). Currently, the synthetic approaches of SeNPs mainly include physical, chemical, and biological methods. Taken together, biogenic synthesis of SeNPs based on probiotic bacteria attracted wide attention due to its unique advantages such as safe, low cost production, low toxicity, and various potential function (Husen and Siddiqi, 2014). Many bacteria possess the ability to transform selenite to elemental Se with less or even no toxicity (Gerrard et al., 1974). Bacteria reduce Se (IV) to SeNPs either under aerobic or anaerobic conditions. In the current study, we found that probiotic *L. casei* 393 effectively transformed sodium selenite to SeNPs under anaerobic conditions, which may be one of the mechanism of Se detoxification by bacteria (Kessi et al., 1999). Previous researches suggest that molecular mechanism of Se (IV) reduction is involved in three different pathways: (1) the periplasmic nitrite reductase (DeMoll-Decker and Macy, 1993); (2) redox precipitation of both elemental sulfur and elemental Se (Hockin and Gadd, 2003); (3) a glutathione (GSH) reductase catalyzes the reaction of GSH with Se (IV) to produce GS–Se–SG, further generate GS–Se (Hunter, 2014). Periplasmic nonspecific selenite reductases are involved in reduction of selenite to SeNPs. These reductases mainly include nitrite reductase, sulfite reductase, and GSH reductase (Butler et al., 2012). However, the exact biogenic synthesis mechanism of SeNPs by *L. casei* 393 remains unclear. The synthetic position of SeNPs could be extracellular, intracellular, or membrane bound (Wadhwani et al., 2016). The synthetic position may be different from the accumulation position. Biogenerated SeNPs accumulate in the bacterial cell during mid- to late-exponential growth phases and secreted into the surrounding medium in a stationary phase (Butler et al., 2012). In the study, *L. casei* 393 accumulated biogenic SeNPs intracellularly, and the particle size was 50–80 nm. However, previous research indicate that the size of the nanoparticles produced by *L. casei* is 150–400 nm, and *L. acidophilus* produces lactomicroSel particles with a size of 200–350 nm (Nagy et al., 2016). We speculate that different microorganism and synthetic conditions may affect the particle size of biogenerated SeNPs. The assembly of Se nanosphere is closely associated with proteins (Kaur et al., 2009). Proteins with tyrosine, tryptophan, and/or phenylalanine residues play a crucial role in SeNPs synthesis (Li et al., 2007).

Selenium is a nutritionally essential trace element with antioxidant properties. In the present study, the antioxidant activities of *L. casei* 393-SeNPs were investigated. We found that pretreatment with *L. casei* 393-SeNPs effectively attenuated diquat-induced oxidative damage in porcine intestinal epithelial cells (IECs). Moreover, compared with the model group, *L. casei* 393-SeNPs reduced the level of MDA in culture medium, and up-regulate protein expression levels of ZO-1, occluding, and claudin-1. SeNPs-loaded chitosan microspheres possessed powerful antioxidant activities, and significantly increased Se retention and levels of GPx, SOD, and catalase (CAT) (Bai et al., 2017). Probiotics can be used as an adjuvant for cancer prevention and/or treatment through their abilities to modulate intestinal microbiota and host immune response (So et al., 2017). SeNPs possess anticancer activity against kidney, breast, lung, and osteosarcoma (Ali et al., 2013; Ramamurthy et al., 2013; Yazdi et al., 2013). Administration with SeNPs-enriched *Lactobacillus plantarum* decreased the tumor volume and improved the survival rate in test mice compared with *L. plantarum* treatment alone or control group (Yazdi et al., 2012). Anisomycin-loaded functionalized SeNPs induced the cell apoptosis through activating the caspase cascade signaling in HepG2 cells (Xia et al., 2015). The current result indicated that *L. casei* 393-SeNPs effectively induced the apoptosis of HepG2 cells. However, *L. casei* 393-SeNPs exhibit no cytotoxicity on IPEC-J2, NCM460, and THP-1 cells. The antitumor effect of Se is not a single mechanism, but with multiple toxicological effect (Weekley and Harris, 2013). The response of tumor cells to SeNPs may be different from normal cells due to the microenvironment of tumor cells and normal cells growth are different. Hence, the concentration causing toxicity to the tumor cells and normal cells is different. On the other hand, the receptors-mediated antitumor effect of *L. casei* 393-SeNPs is different. The anticancer properties depend on the Se species, dose, cancer type, and stage (Etminan et al., 2005), which can be affected by environmental factors, genotype, and bioavailability of Se.

The intestinal epithelial barrier plays a key role in preventing pathogen invasion and maintaining intestinal health. IECs are an important part of maintaining the barrier integrity and function. Previous research indicated that probiotics are able to modulate the functions of IECs and antagonize the pathogenic bacterium (Qiu et al., 2017; Zeng et al., 2017). However, the protective effect and mechanism of *L. casei* 393-SeNPs remains unclear. Although numerous therapeutic measures such as antibiotherapy, gastrointestinal infection remains a series of problems. ETEC strains responsive for diarrhea are one of the causes of GI infections. In swine, ETEC is responsible for neonatal and postweaning diarrhea. On the other hand, pathogenic bacteria infection-caused inflammatory response is often accompanied by oxidative stress. In the current study, the obtained result suggested that the intestinal barrier dysfunction caused by ETEC K88 was ameliorated effectively by *L. casei* 393-SeNPs, the mechanism of which may be related to the antioxidant activity and the modulation of IECs permeability, intestinal epithelial tight junction proteins, and cell signaling pathway mediated by pattern recognition receptors (PRRs). Probiotic bacteria alter PRRs expression and cytokine profile in a human macrophage model challenged with candida albicans and lipopolysaccharide (Matsubara et al., 2017). In addition, oral administration with *L. casei* 393-SeNPs effectively protects ETEC K88-induced intestinal injury. The possible mechanism may be involved in maintaining the integrity of intestinal epithelial barrier function and balance of intestinal microflora. Moreover, *L. casei* 393-SeNPs treatment affected the expression of BDNF and TrkB, which suggest that gut–brain axis is involved in the protective effect of *L. casei* 393-SeNPs. Our findings were consistent with previous reports. *L. casei* DN-114 001 inhibits the increase in enteropathogenic *Escherichia coli* (EPEC)-induced paracellular permeability (Parassol et al., 2005). *L. casei* prevents cytokine-induced epithelial barrier dysfunctions in IECs (Eun et al., 2011). These properties could partly explain the health benefits of probiotics for host defenses capabilities, such as associated with prevention of diarrhea.

## Conclusion

Probiotic bacteria *L. casei* 393 reduced toxic Se (IV) to non-toxic red SeNPs with sizes ranging from 50 to 80 nm under sodium selenite stress and anaerobic conditions. Moreover, *L. casei* 393 accumulated biogenic SeNPs intracellularly. *L. casei* 393-SeNPs induced HepG2 cells apoptosis and improved diquat-caused oxidative damage in IECs and alleviated ETEC K88-caused intestinal barrier dysfunction through exhibiting antioxidant activity, regulating inflammatory response, and maintaining intestinal epithelial barrier integrity and intestinal microflora balance. These findings provide an important reference for developing no-toxic Se supplement agents and microecological agents with diverse functions.

## Author Contributions

All authors listed have made a substantial, direct, and intellectual contribution to the work, and approved it for publication. CX and YG designed the overall research and wrote the paper.

## Conflict of Interest Statement

The authors declare that the research was conducted in the absence of any commercial or financial relationships that could be construed as a potential conflict of interest.
